# The effect of female breast surface area on cutaneous thermal sensation, wetness perception and epidermal properties

**DOI:** 10.1113/EP092158

**Published:** 2024-11-29

**Authors:** Hannah Blount, Alessandro Valenza, Jade Ward, Silvia Caggiari, Peter R. Worsley, Davide Filingeri

**Affiliations:** ^1^ ThermosenseLab, Skin Sensing Research Group, School of Health Sciences The University of Southampton Southampton UK; ^2^ Sport and Exercise Sciences Research Unit, SPPEFF Department University of Palermo Palermo Italy; ^3^ PressureLab, Skin Sensing Research Group, School of Health Sciences The University of Southampton Southampton UK

**Keywords:** breast, female, morphology, skin, thermal sensation, wetness perception

## Abstract

Female development includes significant size changes across the breast. Yet, whether differences in breast surface area (BrSA) modify breast sensitivity to warm, cold and wetness, and the associated epidermal properties (skin thickness and surface roughness) remain unclear. We investigated the relationship between BrSA and thermal and wetness perception, as well as epidermal properties, in 21 females (28±10 years) of varying breast sizes (BrSA range: 147–502 cm^2^), at multiple breast sites (i.e., nipple, above and below the nipple, and bra triangle). Associations between BrSA and the perceptual and epidermal variables were determined via correlation analyses. Differences across test sites were assessed by repeated‐measures ANOVA. Our results did not support the hypothesis that larger breasts present reduced thermal and wetness sensitivity, except for the above nipple site, which presented reduced warm sensitivity with increasing BrSA (*r* = −0.61, *P* = 0.003). We also found a heterogeneous distribution of cold, but not warm or wetness, sensitivity across the breast, with the above nipple site presenting lower cold sensitivity than any other site (*P* < 0.015). Our findings did not indicate any association between BrSA and epidermal properties (thickness and roughness), nor any site‐dependent variation in these anatomical parameters (*P* > 0.15). We conclude that, while some skin‐site (i.e., above the nipple) and perceptual modality‐dependent (i.e., warm sensitivity) differences were observed, BrSA‐dependent variations in thermal and wetness sensitivity were not a generalised feature of the skin covering the breast. These observations advance our fundamental understanding of breast sensory function, and they could inform the design of user‐centred clothing such as bras.

## Abstract (highlights)

- **What is the central question of this study?**
  How do differences in breast surface area (BrSA) impact thermal sensation, wetness perception and epidermal properties across the female breast?
- **What is the main finding and its importance?**
  While some skin‐site (i.e., above the nipple) and perceptual modality‐dependent (i.e., warm sensitivity) differences were observed, BrSA‐dependent variations in thermal and wetness sensitivity were not a generalised feature of the skin covering the breast. These observations advance our fundamental understanding of breast sensory function, and they could inform the design of user‐centred clothing such as bras.

## 1 INTRODUCTION

The ability to sense changes in body temperature and to consciously experience thermal sensations is a fundamental cutaneous sensory attribute that is necessary for optimal interactions with our surrounding thermal environment, as well as with the objects that contact our skin, such as clothing (Havenith, 2002; Song, 2011). The perception of skin wetness, which is a synthetic perception arising from the integration of thermal and tactile cues originating at the skin when this is wetted (Filingeri et al., 2014), also impacts the subjective experience of skin–clothing interactions, which can in turn lead to thermal and clothing discomfort (Gwosdow et al., 1986).

Empirical evidence indicates that innate differences may exist between men and women in their skin thermal and wetness sensitivity (Greenfield et al., 2023). For example, women have been shown to often report more intense thermal sensations than men for the same absolute temperature (Gerrett et al., 2014; Inoue et al., 2016; Li et al., 2008), as well as to present greater sensitivity to cold‐wet stimuli (Valenza et al., 2019). However, it has also been proposed that sex‐related differences in local thermal and wetness perceptions could be partly explained by differences in body morphology (Filingeri et al., 2025). Specifically, women often present a smaller (on average) body surface area (BSA) than men, and this may in turn result in a larger relative proportion of their BSA being stimulated by fixed‐size thermal stimuli (Filingeri et al., 2018; Luo et al., 2020).

While the evidence above may (partly) explain some previously reported thermal sensitivity differences between men and women, it also opens to the question of how differences in body morphology within a specific group, such as women, may impact on individual responses to cutaneous thermal and wet stimuli. This question is relevant, as women undergo unique anatomical, physiological and hormonal changes across the lifespan that impact their body morphology. For example, consider the impact of sexual maturation on breast development and the resulting breast size, which can vary greatly due to genetic factors, body mass index, and energy intake early in life (Trichopoulos & Lipman, 1992; Wade et al., 2010).

Variations in breast size lead to differences in breast surface area (BrSA), which could potentially translate to varying densities of thermoreceptors innervating the skin of the breast (Adair, 1999). It is indeed well established that the nervous system reaches maturity prior to breast development (Javed & Lteif, 2013). Hence, one may hypothesise that breasts with greater BrSA may present reduced density of thermoreceptors, and potentially lower thermal sensitivity (Moini et al., 2021). However, knowledge on how thermal and wetness sensitivity might differ amongst women varying in BrSA and the implications that this might have for female‐specific clothing comfort (e.g., in the context of wearing bras) continue to be lacking.

The available evidence on breast thermal sensitivity often lacks systematic measurements of (and/or control for) breast size and BrSA. For example, Luo et al. (2020) has recently reported a homogeneous distribution of thermal sensitivity across the breast. By contrast, other studies have reported a more heterogeneous thermal sensitivity distribution (Terzis et al., 1987; Valenza et al., 2023). Specifically, Valenza et al. (2023) demonstrated regional variation in warm thermal sensation with significantly greater sensitivity in the lower breast region compared to bra triangle, yet they showed no regional variation in response to cold thermal stimulation. Furthermore, an uneven distribution of thermal sensitivity was demonstrated by Terzis et al. (1987) who observed the lateral surface of the breast to demonstrate greater thermal sensitivity than the areola. These contrasting results have contributed to a certain level of ambiguity in the exact patterns of regional sensitivity across the skin of the female breast, particularly when this varies in size and surface area.

Regarding wetness perception, there is a paucity of evidence on how this may vary across the breast. Evidence is available on breast sensitivity to tactile stimuli, and this has indicated that larger breasts tend to have lower tactile sensitivity (Cornelissen et al., 2018; DelVecchyo et al., 2004; Tairych et al., 1998) and spatial acuity (Long et al., 2022). Owing to the dependence of wetness perception on thermo‐tactile inputs, it could be hypothesised that size‐dependent differences in thermal or tactile sensation may translate to a size‐dependent difference in wetness perception. Yet, this hypothesis is yet to be tested empirically.

Variations in breast size BrSA can also lead to changes in breast skin stretch, with consequent implications for the mechanical properties of the breast skin. Acute skin stretching has been shown to reduce skin surface roughness by up to 50% (Maiti et al., 2016). Furthermore, load‐bearing skin regions have demonstrated greater epidermal thickness compared to non‐load‐bearing sites (Lundström et al., 2018; Maiti et al., 2020). When considering females with greater breast volume and mass, the supporting skin tissue will be exposed to greater load than in females with smaller breasts. However, it remains unknown how the effect of breast size may impact the epidermal properties of breast skin.

When modelling factors governing thermal sensation beyond the thermal characteristics of the skin and of the contacting material, evidence indicates that the mechanical properties of the skin could also influence heat exchange and the resulting thermal sensation upon contact with materials (Chen & Ding, 2019). For example, skin characteristics at the fingertip, namely epidermal thickness and surface roughness, have been shown to impact thermal conductance. As surface roughness and thickness increase, there is greater thermal contact resistance, with a reduction in heat flux to the dermal tissues (Chen & Ding, 2019). Thus, differences in BrSA and the associated skin remodelling could lead to changes in thermal conductance, which could in turn impact local thermal sensitivity across breast sizes. However, whether local variations in epidermal properties across breasts of varying size contribute to individual differences in local thermal sensitivity remains under investigated.

Breast thermal sensations play an important role in female thermal comfort at rest (Ayres et al., 2013). Furthermore, the perception of wetness will impact on perceptions of clinginess and comfort in this unique part of the female body, which is almost always covered by a bra (Song, 2011). Hence, broadening our fundamental understanding of the role of breast size and BrSA on thermal and wetness sensitivity, and on skin properties, could inform the design of user‐centred garments such as bras that help improve the comfort of individuals with varying breast sizes.

The primary aim of this study was to investigate the relationship between BrSA and thermal and wetness perceptions over various breast regions in a cohort of healthy young to middle‐aged females. We hypothesised that larger breasts would present reduced thermal and wetness perception than smaller breasts. The secondary aim of this study was to investigate the relationship between BrSA and epidermal properties in the same breast regions. We hypothesised that increased breast size would result in an increase in epidermal thickness, with potential implication for local thermal sensitivity.

## 2 METHODS

### 2.1 Ethical approval

This study was approved by the University of Southampton Ethics Committee (approval no. 79007). All participants provided written informed consent prior to testing. The study conformed to the ethical standards set by the *Declaration of Helsinki*.

### 2.2 Participants

This study used a purposeful sampling approach to recruit females with varying BrSA in the local community. Due to the non‐linear association between BrSA and bra size (i.e., the latter being the most intuitive way of determining one's breast size for eligibility purposes) we opted for the recruitment of four to six females for each of four bra‐size categories, namely small (32C–32E and 34A–34C), medium (34D–34E and 36A–36C), large (36D–36E and 38A–38C), and extra‐large (38D–38E and 40A–40C). Females are typically familiar with such a classification of bra size, and this was used to aid participant recruitment. A total sample size of 20–24 healthy young and middle‐aged females were targeted, which has been shown to be sufficient in human thermoregulation studies (Buono & Connolly, 1992; Havenith et al., 2008; Kondo et al., 2001).

A total of 21 females were recruited to the study (age: 27.7 ± 9.6 years; weight: 72.2 ± 12.7 kg; height: 170.4 ± 4.8 cm) (Table 1). Inclusion criteria included physically active females (i.e. performing 30 min regular exercise of moderate intensity at least 3 days each week), free from musculoskeletal or neurological disease, not under any pharmacological treatment, with standard breast tissue type (i.e. no implants, reductions or mastectomy). They were also instructed to refrain from: (1) performing strenuous exercise in the 48 h preceding testing; (2) consuming caffeine or alcohol in the 24 h preceding testing; (3) consuming food in the 3 h prior to testing; and (4) applying creams or gels to the chest region. Menstrual phase was not ‘controlled for’ based on preliminary evidence that thermal sensation in females may not be independently modified by menstruation (Matsuda‐Nakamura et al., 2015). However, self‐reports of menstrual phase were collated. Nineteen participants were spread across a typical 28‐day menstrual cycle (mean day of cycle: 13.6 ± 8.2) and two participants presented irregular periods at the time of the study.

**TABLE 1:** Participant demographics (*n* = 21).

	Age (years)		Height (m)		Weight (kg)		BrSA (c $m^{2}$)		Proportional area of stimulated skin relative to BrSA (%)		BSA ($m^{2}$)		Proportional area of stimulated skin relative to BSA (%)	
**Bra Size**	Mean	Min–max	Mean	Min–max	Mean	Min–max	Mean	Min–max	Mean	Min–max	Mean	Min–max	Mean	Min–max
**Small (*n* = 6)**	23.3	18–30	1.67	1.63–1.72	60.8	56.4–68.9	168.0	147.2–230.1	15.2	10.9–17.0	1.68	1.60–1.75	0.15	0.14–0.16
**Medium (*n* = 5)**	22.8	19–27	1.69	1.65–1.70	67.5	59.8–76.1	246.3	203.5–288.0	10.3	8.7–12.3	1.77	1.66–1.87	0.14	0.13–0.15
**Large (*n* = 6)**	30.2	20–42	1.72	1.68–1.77	72.3	61.3–83.4	316.0	173.7–402.2	8.5	6.2–14.4	1.85	1.70–1.94	0.14	0.13–0.15
**X‐large (*n* = 4)**	29.8	21–44	1.75	1.66–1.83	94.1	87.0–97.9	432.7	300.0–502.2	6.0	5.0–8.3	2.09	1.88–2.21	0.12	0.11–0.12

Mean, minimum and maximum values. BrSA, breast surface area; BSA, body surface area.

To consider the potential effect of spatial summation (Courtin et al., 2023) in the investigation of thermal and wetness perception, it is necessary to account for the fixed‐size stimulus probe (25 cm^2^) and associated area of stimulated skin. Therefore, the proportional area of stimulated skin relative to BrSA and BSA was calculated following these equations:

$$\mathrm{Proportion}\;\mathrm{BrSA}\left(\%\right)=\frac{25}{\mathrm{BrSA}\;(cm^{2})}\times 100$$

$$\mathrm{Proportion}\;\mathrm{BSA}\left(\%\right)=\frac{0.0025}{\mathrm{BSA}\;(m^{2})}\times 100$$

where BSA was calculated as:

BSA = Wt (kg)^0.425^ × Ht (cm)^0.725^ × 0.007184 (Du Bois & Du Bois, 1916)

Proportional coverage relative to BrSA and BSA are reported in Table 1. It can be observed that while the proportional area of stimulated skin relative to BrSA decreased from smaller to larger breasts (i.e., from ∼15% to ∼6%), this translated into a much smaller difference between smaller and larger breasted females when expressed as proportional area of stimulated skin relative to BSA (i.e. from ∼0.15% to ∼0.12%).

### 2.3 Experimental design

**FIGURE 1:**
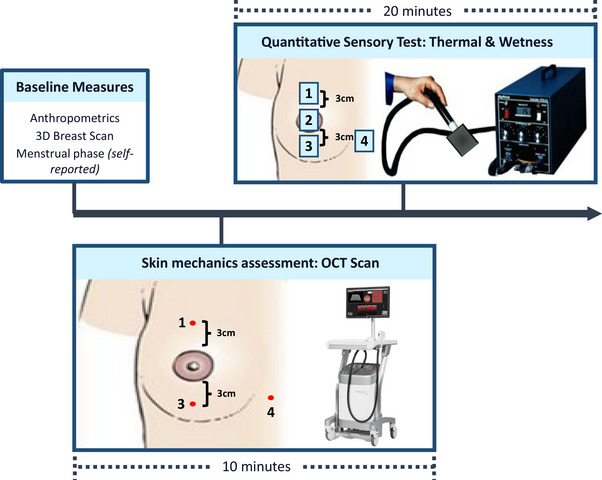
Schematic representation of experimental design and tested sites across the breast and bra triangle.

To establish breast size‐dependent differences in thermal and wetness perception, participants visited the lab on one occasion in a quasi‐experimental study design. Firstly, BrSA geometry was captured, then epidermal measures and thermal and wetness sensitivity were assessed at rest. This included multiple breast locations longitudinally down the breast, in line with the nipple (Figure 1). Spatial perception mapping was deemed important to capture potential regional differences in skin properties, as previously noted in the case of breast thermal sensitivity (Valenza et al., 2023). Evaluation of epidermal properties was also deemed important to provide insights into the potential causes of perceptual variation, as skin thickness and structure have been shown to impact the rate of heat transfer and to elevate perception thresholds (Chen & Ding, 2019; Lundström et al., 2018).

#### 2.3.1 Thermal and wetness perception

A single‐blind psychophysical approach based on a well‐established quantitative sensory test (QST) of skin thermal and wetness sensing (Valenza et al., 2019) was used. This enabled mapping of differences in regional thermal and wetness sensitivity at rest in a thermoneutral environment (21 ± 1.5°C and 37± 5.2% relative humidity). The QST involved participants having to report the perceived magnitude of local thermal sensations and wetness perceptions arising from a short‐duration (i.e. 5 s) static application of cold‐wet (i.e. 5°C below local skin temperature, *T*_skin_) or warm‐wet (i.e. 5°C above local *T*_skin_) stimuli delivered with a hand‐held, temperature‐controllable probe (NTE‐2A, Physitemp Instruments, Clifton, NJ, USA; surface area: 25 cm^2^; water content: 0.8 mL). Participants reported the extent of their local perceptions using two digital visual analogue scales (VAS) for thermal sensation (length 200 mm; anchor points: 0, very cold; 100, neutral; 200, very hot) and wetness perception (length: 100 mm; anchor points: 0, dry; 100, completely wet). The study employed thermal stimuli relative to the local *T*_skin_ to account for inter‐individual variability in local *T*_skin_, such that the same relative thermal stimulus was applied to all participants (Darian‐Smith & Johnson, 1977).

Sensitivity was mapped at three locations over the right breast (above the nipple, nipple–areola complex, below the nipple) and bra triangle (Figure 1). These locations were selected because: (1) the nipple is thought to be the centre of breast sensitivity (Long et al., 2022), and (2) thermal sensitivity differences have been shown to be greatest in the longitudinal plane on the breast (Valenza et al., 2023). As with previous studies (Filingeri et al., 2014, 2018; Valenza et al., 2023), participants were only informed of the location of stimulation and blinded to the nature of the stimuli to limit expectation biases. Moreover, participants underwent a systematic familiarization to the testing procedures and perceptual scales prior to testing (Valenza et al., 2019). The same investigator performed all testing to ensure internal consistency.

#### 2.3.2 Epidermal measures

Epidermal properties of thickness and surface roughness were evaluated non‐invasively using an optical coherence tomography (OCT) scanner (Vivosight, Michelson Diagnostics Ltd, Weavering, UK). The VivoSight is a Fourier domain OCT system which captures image data at 20 Hz. The OCT image volume obtained from each skin site was 6 × 6 × 2 mm^3^ (width × length × depth). Epidermal properties were assessed at three locations that corresponded to the sites of sensitivity measurements. These properties were estimated using the proprietary software associated with the imaging system (VivoTools, Michelson Diagnostics Ltd).

### 2.4 Experimental procedures

Upon arrival to the laboratory, participants completed a questionnaire to report estimated menstrual phase and contraceptive use. Anthropometric measures of height, weight and BrSA were taken in a thermoneutral laboratory (21 ± 1.5°C and 37± 5.2% relative humidity). Height was measured on a wall stadiometer and weight on a precision scale (KERN 150K2DL, Balingen, Germany). Participants were then asked to remove their bra and position themselves in a 4‐point prone position. BrSA was then estimated using a three‐dimensional (3D) white‐light surface scan (EinScan H, Shining 3D Tech. Co. Ltd., Hangzhou, China) with a calibrated surface height accuracy of 0.05 mm. Markers were placed around the breast border based on a validated breast volume model (Göpper et al., 2020), from which surface area was extracted using MeshLab (Visual Computing Lab, CNR‐ISTI, Pisa, Italy). This allowed a 15‐min period of rest to adjust to the internal environmental conditions.

**FIGURE 2:**
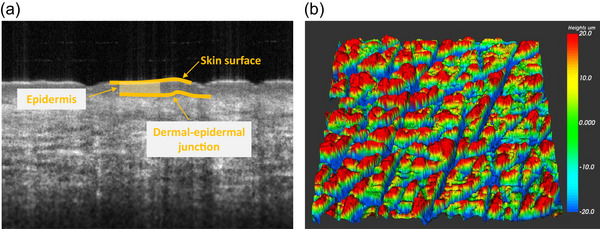
(a) Annotated 2D‐OCT scan of the breast skin. (b) 3D surface roughness scan with scale.

Following the 3D scan, participants were asked to lie in a supine position and the epidermal measurements were taken using the OCT scanner. Test sites were marked with a washable marker and participants were familiarised with the procedure. Figure 2 shows an example of an OCT scan. Clearly visible are features such as the epidermis and dermis. Upon completion of the OCT imaging, thermal and wetness perception were assessed. Participants were familiarised with the experimental procedures and VAS for the thermal and wetness QST. The local *T*_skin_ was recorded with an infrared camera for each test site (ER53, FLIR Systems, Wilsonville, OR, USA). A 100% cotton fabric patch was applied to the thermal probe, which was then wetted with 0.8 mL of water using a pipette to ensure full saturation. The order of stimulus modality (i.e., warm vs. cold) and site (i.e., skin region) was randomised to minimize order effects. A verbal warning was given, and the stimulus applied for 5 s, during which the participant was asked to rate their immediate thermal and wetness perception using the VAS. Probe application pressure was not measured but was controlled to be sufficient to ensure full contact without causing pronounced skin indention. Following the submission of the perceptual rating, the stimulus was removed, skin dried and local *T*_skin_ was recorded again, before proceeding to the next skin site.

### 2.5 Statistical analysis

Data normality was assessed using the Shapiro–Wilk test. Thermal and wetness sensitivity and surface roughness were identified to be normally distributed, and hence parametric tests were used for analysis. Epidermal thickness data were identified to be non‐normally distributed, so non‐parametric descriptors and tests were applied. Statistical analysis was completed using the SPSS Statistics package (version 28.1, IBM Corp., Armonk, NY, USA) with significance set at *P* < 0.05.

We assessed the relationship between BrSA with thermal sensation and wetness perception, as well as with epidermal measures using Pearson's correlation and Spearman's rank correlation analyses, respectively. Correlation coefficients were calculated separately for each of the skin sites tested.

Regional variations in thermal sensation and wetness perception across the breast were analysed separately for the effects of stimulus temperature (2 levels, i.e., warm and cold) and skin site (4 levels) by means of two‐way repeated measures ANOVA. Regional variation in surface roughness was analysed using a repeated measures ANOVA (3 levels; skin site). To investigate site effects on epidermal thickness, Friedman's test was used. In the event of statistically significant main effects, *post hoc* analyses were conducted with Bonferroni correction.

To explore inter‐individual variability in the sensory outcomes (thermal and wetness perception) and epidermal measures, coefficient of variations (i.e., (SD/mean) × 100) were calculated for each skin site and stimulus modality (cold‐thermal (CT), cold‐wetness (CW), hot‐thermal (HT), hot‐wetness (HW)). Data were summarized into heat maps to display skin locations of the breast with high and low inter‐subject variability.

To determine whether any regional differences in perception were related to any changes in epidermal properties, Pearson's correlation analyses were performed between each stimulus modality (CT, CW, HT, HW) and epidermal outcome (epidermal thickness, surface roughness) at the three cross‐over test sites (above nipple, below nipple, bra triangle).

## 3 RESULTS

### 3.1 Breast size‐dependent differences in thermal and wetness perception

**FIGURE 3:**
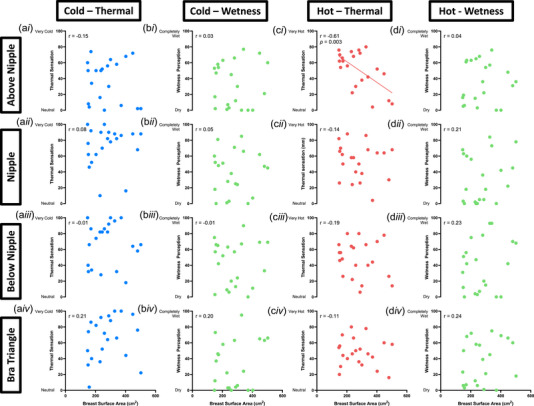
Relationships between breast surface area and four stimuli (cold‐thermal (a), cold‐wet (b), hot‐thermal (c), hot‐wet (d) at four skin sites (above nipple (i), nipple (ii), below nipple (iii), bra triangle (iv)) (*n* = 21).

The relationships between BrSA with thermal sensation and wetness perception at all skin sites are presented in Figure 3. The data analysis indicated that the only statistically significant correlation occurred between BrSA and warm sensation at the above the nipple site (Pearson *r* = −0.61, *P* = 0.003). No other statistically significant correlation was identified for any other skin site or perceptual modality. (*P* > 0.12).

### 3.2 Regional variations in thermal sensation and wetness perception

**FIGURE 4:**
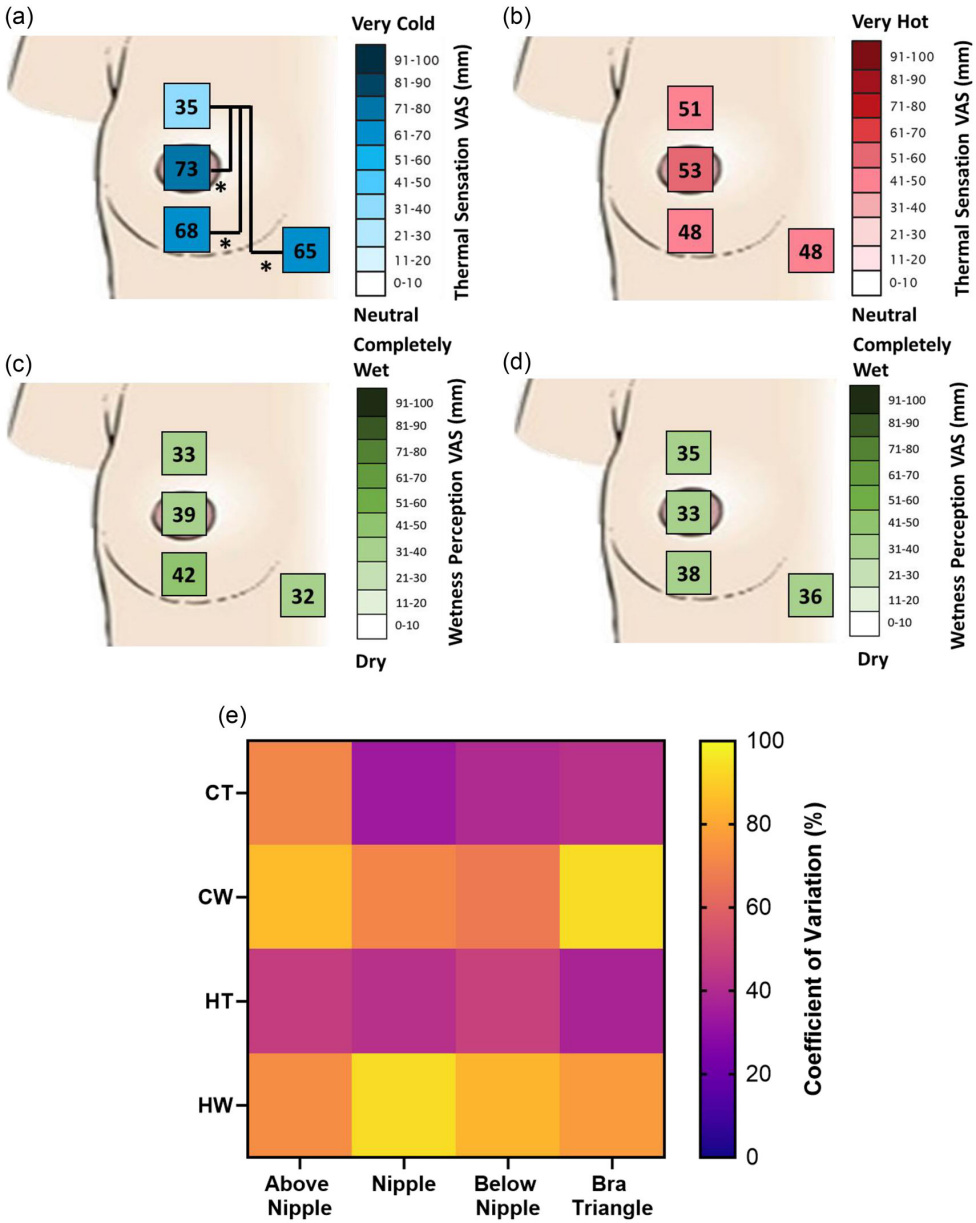
Mean (*n* = 21) cold‐thermal (a), hot‐thermal (b), cold‐wet (c), and hot‐wet (d) sensitivity for the four locations tested. Statistically significant multiple comparisons are pictured (*P < 0.05). (e) Coefficient of variations (%) at each test site for cold‐thermal (CT), cold‐wet (CW), hot‐thermal (HT) and hot‐wet (HW) sensations.

Regarding thermal sensation, there was a statistically significant effect of skin site (*F*(3, 60) = 6.07; *P* = 0.001), no effect of stimuli temperature (*F*(1, 20) = 4.07; *P* = 0.057), and an interaction between skin site and stimuli temperature (interaction: *F*(3, 60) = 8.3; *P* < 0.001). Specifically, it was found that cold (Figure 4a), but not warm (Figure 4b), sensations varied across skin sites, such that less intense cold sensations were reported when stimulating the above nipple site (35.1 ± 28.7 mm) than the nipple (72.7 ± 24.6 mm; *P* < 0.001), below nipple (67.5 ± 26.9 mm; *P* = 0.014), and bra triangle sites (65.2 ± 27.8 mm; *P* = 0.010).

Regarding wetness perception, no significant effect of skin site (*F*(3, 60) = 0.90; *P* = 0.445), stimuli temperature (*F*(1, 20) = 0.11; *P* = 0.747) or interaction (*F*(3, 60) = 0.73; *P* = 0.538) was found at any test site (Figure 4c, d).

With respect to inter‐individual variability in perceptual responses (Figure 4e), we found this to vary from: (1) a minimum of 34% (nipple) to a maximum of 71% (above the nipple) for cold sensations; (2) a minimum of 37% (bra triangle) to a maximum of 48% (below the nipple) for warm sensations; (3) a minimum of 67% (below the nipple) to a maximum of 94% (bra triangle) for cold‐wet perceptions; and (4) a minimum of 73% (above the nipple) to a maximum of 93% (nipple) for warm‐wet perceptions.

### 3.3 Breast size‐dependent differences in epidermal measures

**FIGURE 5:**
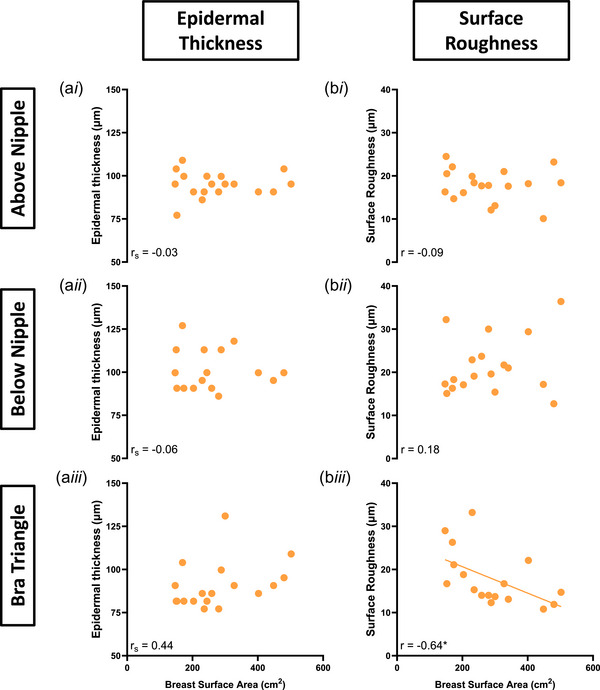
Relationships between breast surface area and epidermal measurements (epidermal thickness (a), surface roughness (b)) at three skin sites (above nipple (1), below nipple (2), bra triangle (3)) (*n* = 21). r, Pearsons correlation; r_s_, Spearmans correlation (non‐parametric).

The relationships between BrSA with epidermal thickness and surface roughness at all skin sites are presented in Figure 5. Correlation analyses between BrSA and epidermal thickness indicated no statistically significant associations at any breast sites (*P *≥ 0.195). A statistically significant negative correlation between BrSA and skin roughness was found only at the bra triangle (Pearson *r* = −0.64, *P* = 0.005).

### 3.4 Regional variations in epidermal measures

**FIGURE 6:**
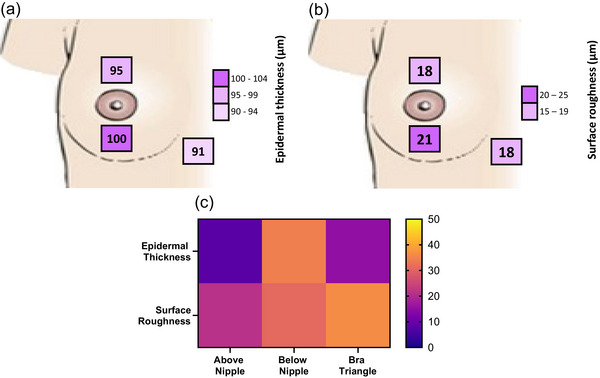
Mean (*n* = 21) (a) epidermal thickness, (b) surface roughness at the three skin sites. Statistically significant comparisons are pictured. (c) Coefficient of variations (%) at each test site for epidermal thickness and surface roughness.

There was no significant effect of test site on epidermal thickness (*P* = 0.421, Figure 6a) nor on surface roughness (*P* = 0.158, Figure 6b).

Inter‐individual differences in epidermal thickness ranged from 8% (above the nipple) to 34% (below the nipple), while variability in surface roughness ranged from 21% (above the nipple) to 36% (bra triangle) (Figure 6c).

### 3.5 Relationship between perception and epidermal properties

The correlation coefficients between epidermal properties and thermal sensation and wetness perception at all skin sites are presented in Table 2. Overall, no significant associations were found between epidermal thickness and surface roughness with any perceptual variable at any test site.

**TABLE 2:** Pearson correlation coefficients and *P*‐values between epidermal properties with thermal sensation and wetness perception at all skin sites.

		Cold thermal		Cold wetness		Hot thermal		Hot wetness	
		*r*	*P*	*r*	*P*	*r*	*P*	*r*	*P*
**Above nipple**	Epidermal thickness	0.13	0.614	0.23	0.373	−0.15	0.559	0.17	0.527
	Surface roughness	−0.35	0.159	−0.37	0.136	−0.01	0.955	−0.24	0.347
**Below nipple**	Epidermal thickness	0.23	0.380	−0.29	0.263	−0.04	0.888	−0.28	0.269
	Surface roughness	−0.16	0.538	−0.19	0.455	0.16	0.649	−0.11	0.659
**Bra triangle**	Epidermal thickness	0.16	0.552	−0.09	0.728	0.04	0.887	−0.45	0.067
	Surface roughness	−0.47	0.057	−0.22	0.398	0.42	0.095	−0.34	0.178

## 4 DISCUSSION

The primary aim of this study was to investigate the relationship between BrSA and thermal sensation and wetness perception over various breast regions. Our findings did not support the hypothesis that skin sites across larger breasts present reduced thermal and wetness sensitivity, except for the site above the nipple, which instead exhibited a reduction in warm sensitivity with increasing BrSA. Our findings also indicated a heterogeneous distribution of cold, but not warm nor wetness, sensitivity across the breast, with the site above the nipple presenting lower cold sensitivity than any other tested site. Regarding our secondary hypothesis, our findings did not indicate any association between BrSA and epidermal properties (i.e. thickness and roughness), nor did they indicate any site‐dependent variation in these anatomical parameters. Finally, the lack of any meaningful association between any perceptual response and their site‐specific epidermal parameters indicated that the observed body‐regional and inter‐individual variability in sensitivity is unlikely to be dependent on epidermal parameters. These observations are discussed in detail in the sections below.

### 4.1 BrSA‐ and skin‐site‐dependent differences in perception

The relationship we observed between increasing BrSA and decreasing warm sensitivity at the site above the nipple is interesting and it may be underlined by a size‐dependent variation in the density of warm thermoreceptors with spot‐like receptive field (Kenshalo & Gallegos, 1967) that may innervate the skin of larger breasts. This hypothesis is not entirely speculative, if one considers that the nervous system reaches maturity prior to breast development (Javed & Lteif, 2013). Furthermore, this hypothesis would align with our previous findings of a reduced sweat gland density at the breast with increasing BrSA in the same cohort of females tested in the present study (Blount et al., 2024a). A size‐dependent relationship between tactile sensitivity and BrSA has also been previously reported, and it demonstrated that larger breasts tend to have overall lower tactile sensitivity (Cornelissen et al., 2018; DelVecchyo et al., 2004; Tairych et al., 1998) and spatial acuity (Long et al., 2022) than smaller breasts (note this effect is particularly evidence at the upper and lateral breast regions) (Tairych et al., 1998). However, it is important to note that we recently failed to replicate these mechano‐sensory findings when stimulating the lower breast of the same cohort of females tested in the present study (Blount et al., 2024b). These contrasting findings may highlight the heterogeneity of such size‐dependent perceptual mechanisms across skin sites, as well as their individual variability. This is clearly demonstrated by our observation that, out of the 16 possible combinations of perceptual assessments (i.e. cold, warm, cold‐wet, and warm‐wet) by skin site (i.e. above the nipple, nipple–areola complex, below the nipple and bra triangle), a statistically significant size‐dependent association was identified in one instance only (i.e., warm sensitivity at the site above the nipple). Despite the relevance of the warmth‐related findings at the site above the nipple, we therefore believe that a BrSA‐dependent variation in thermal/wetness sensitivity may not be considered a robust and generalised feature of the skin covering the female breast.

Besides size‐dependent differences, it is also worth noting that the site above the nipple was the only region to differ substantially from the other tested sites, by presenting a meaningfully lower thermal sensitivity to cold only. As we did not observe any other site‐by‐perceptual modality difference, one may argue that, albeit varying at an individual level (see Figure 4), thermal and wetness sensitivity across the breast sites tested in this study is rather homogeneous. To date, only a few studies have investigated regional variations in breast thermal sensitivity (Luo et al., 2020; Terzis et al., 1987; Valenza et al., 2023). Our findings somewhat correspond to those of Luo et al. (2020), who found a homogeneous thermal sensitivity distribution across the breast. However, this is in contrast with the findings of Valenza et al. (2023), who demonstrated differences in warm thermal sensitivity between the lower breast and the bra triangle. Furthermore, the lower cold sensitivity at the site above the nipple that we observed was not identified by Luo et al. (2020) or Valenza et al. (2023). While methodological differences amongst these studies may underlie some contrasting results (e.g., Luo et al. (2020) used a thermal probe with a coverage of 1.32 cm^2^ compared to the 25 cm^2^ probe used in this study), these do not apply consistently (e.g., both our study and that of Valenza et al. employed the same size probe). In this respect, inter‐individual variability in study participants (e.g., individuals with varying breast sizes were considered only in our study) as well as in their cold sensitivity at the site above the nipple (see Figure 4), may have also contributed to the heterogeneity of the breast sensorial data reported in the literature. Given the upper breast is being highlighted as a region where perceptual differences may exist relative to BrSA and compared to other chest sites, future studies should investigate what drives the perceptual differences in this area of the breast, particularly as this is commonly under greater tensile strains compared to the lower breast (Norris et al., 2020).

### 4.2 BrSA‐ and skin‐site‐dependent differences in epidermal parameters

No relationship was found between BrSA and epidermal thickness at the breast. Maiti et al. (2020) used an OCT scanner to map skin morphology of 21 skin sites across the body and found epidermal thickness at the chest to average around 93 ± 5 µm, which corresponds to the values found in this study. However, this previous study only contained four females and made no mention of breast size. Work by Sutradhar & Miller (2013) specifically investigated variations in breast epidermal and dermal thickness at 16 breast locations in 23 females using high‐frequency ultrasound. While this study made no mention of breast size, it demonstrated that skin thickness in the medial region was greater than in the lateral region of the breast (*P* = 0.001). Sutradhar & Miller (2013) also found no differences in skin thickness between the superior and the inferior region of the breast (*P* = 0.70), which supports the findings of our study. In contrast, other research has demonstrated that skin thickness (measured using mammography) in the superior breast region was lower than that in the inferior breast region (Ulger et al., 2003; Wilson et al., 1983). Ulger et al. (2003) also observed that skin thickness decreases with increases in age and size in all tested regions. While our study did not infer whether differences in skin thickness are size‐ or age‐driven (Oriba et al., 1996; Waller & Maibach, 2005), it is likely that contrasting findings between the literature and our dataset may be primarily due to differences in measuring techniques (e.g., mammography measurement and OCT techniques present different resolutions of imaging and attenuation of dermal tissues). Future studies should investigate epidermal parameters across all breast regions, in an age‐matched cohorts of females with varying sizes, or BrSA size‐matched cohorts of females of varying ages, to better identify which parameter may play a greater role in individual variation in skin thickness.

Regarding surface roughness, we found no relationship with BrSA, nor skin site variations. Skin stretch has been shown to cause the undulations of the epidermal surface and the epidermal–dermal junction to become flattened (Ferguson & Barbenel, 1981). Skin surface folds provide a reserve of tissue, thus allowing the epidermis to stretch without disrupting the epidermal cells. We had hypothesised that the skin of larger breasts, which supports a greater breast volume and mass, may be in a greater state of stretch, which would in turn have reduced its surface roughness. However, this was not observed. We cannot exclude that this lack of an effect may be partly due to the roughness measurements being collected while participants were lying in a supine posture, which would have reduced gravitational tension at the breasts.

Finally, we found no meaningful association between any perceptual response and their site‐specific epidermal parameters. We believe that this finding may be due to the relatively narrow range of inter‐ and intra‐individual variations observed in epidermal thickness (i.e., 0.08–0.12 mm) and in surface roughness (i.e., 10–33 µm). Indeed, previous evidence has indicated that variations in epidermal thickness in the range of 0.1 to 0.5 mm and in surface roughness in the range of 0.4 to 100 µm may be required to observe a meaningful impact of epidermal thickness and surface roughness on the rate of heat exchange between the material in contact with the skin and the cutaneous thermal receptors that contribute to conscious thermal sensations (Chen & Ding, 2019). As a result, we believe it reasonable to conclude that the observed body‐regional variations in sensitivity observed in this study are more likely to be dependent on inter‐individual variability in the subjective assessment of a thermal stimulus (e.g., lived experiences) than on epidermal parameters.

### 4.3 Limitations

The primary limitation of this study is that we used a stimulating probe of fixed size (i.e., 25 cm^2^) to assess thermal and wetness sensitivity across varying BrSA(s). This approach resulted in a proportional area of stimulated skin relative to BSA ranging between 0.12% and 0.15% (i.e., from the largest to smallest breasted female). We have long known that, given the same local thermal stimulation of the skin, the resulting thermal sensation can vary in magnitude depending on the size of the stimulated area (i.e. the larger the area, the more intense the resulting hot or cold sensation) (Hardy & Oppel, 1937; Stevens et al., 1974). Specifically, previous studies investigating spatial summation in thermal sensitivity have found this phenomenon to occur with differences in proportional area of stimulated skin relative to BSA of as little as 0.04% (Kenshalo & Gallegos, 1967). The difference in proportional area of stimulated skin relative to BSA between the largest (∼0.12% of BSA) and smallest (∼0.15% of BSA) breasted females in the current study corresponded to 0.04% (see Table 1). Hence, it cannot be excluded that this methodological limitation could have somewhat biased our perceptual outcomes towards a scenario where larger breasted females may have reported less intense thermal sensations. However, a BrSA‐dependent relationship was found in only one perceptual modality (warm‐thermal above the nipple). Hence, if a spatial summation bias had occurred in this study, then we would have expected to observe it for all perceptual modalities. It should nevertheless be recognised that an ideal, yet challenging, methodological approach would have been one where a proportional area of stimulated skin relative to BSA is fixed, by increasing the probe size in line with BrSA. Under that scenario, and considering our results (i.e. sensation did not generally vary with BrSA), one may envisage that larger breasted women may have in fact presented greater thermal sensation for the same temperature stimulus. However, this consideration remains speculative, and future studies are therefore warranted to better characterise the complex relationship amongst stimulated skin area, probe size and resulting sensations across individuals varying in body morphology. Furthermore, the implications of using a fixed‐size probe over the nipple–areola complex should also be considered. The nipple–areola complex has been shown to be the anchor of breast sensitivity (Kasielska‐Trojan et al., 2021). In the present study, thermal sensitivity was assessed over the nipple–areola complex when the nipple was not erect, and the probe was placed on the skin such that it would be in full contact with the skin surface. Our qualitative observations indicated that individuals presented different size nipples and areolas. Hence, we cannot exclude that, for example, individuals with larger nipples (i.e., larger area of greater sensitivity) may experience variations in thermal and wetness sensitivity independently of breast surface area. Future studies should therefore consider additional measurements of the size of the nipple–areola complex.

### 4.4 Conclusion

We conclude that BrSA‐dependent variations in thermal and wetness sensitivity are not a generalised feature of the skin covering the female breast, as these appeared to be primarily skin site (i.e., above the nipple) and perceptual modality dependent (i.e., warm sensitivity). Irrespective of BrSA, thermal and wetness sensitivity over the breast did also appear to be rather homogeneous, with only the skin site above the nipple presenting reduced cold sensitivity. Inter‐individual differences may play a greater role in determining such perceptual patterns than epidermal parameters (e.g., skin thickness and roughness), as the latter did not vary either with BrSA or with skin site. Altogether, these observations advance our fundamental understanding of the sensory properties of the female breast. Furthermore, our findings carry applied implications to improve the design and comfort of clothing items such as bras, which may account for the observed interaction between breast size, warm sensitivity and regional differences (i.e., upper vs. lower breast) at rest.

## AUTHOR CONTRIBUTIONS

Hannah Blount, Davide Filingeri and Peter Worsley conceived the initial outline for the article. Acquisition was completed by Hannah Blount, Alessandro Valenza and Jade Ward. Hannah Blount, Silvia Caggiari, Davide Filingeri, and Peter Worsley contributed to interpretation. Hannah Blount, Davide Filingeri, and Peter Worsley contributed to drafting the manuscript. All authors approved the final version of the manuscript and agree to be accountable for all aspects of the work in ensuring that questions related to the accuracy or integrity of any part of the work are appropriately investigated and resolved. All persons designated as authors qualify for authorship and only those who qualify for authorship are listed.

## CONFLICT OF INTEREST

None declared.

## Data Availability

Data will be made available upon publication at the University of Southampton data repository (PURE; URL to be activated upon publication).
